# Hepatitis B Core IgM antibody (anti-HBcIgM) among hepatitis B Surface antigen (HBsAg) negative blood donors in Nigeria

**DOI:** 10.1186/1743-422X-8-513

**Published:** 2011-11-10

**Authors:** Margaret Oluwatoyin Japhet, Olufisayo Adeyemi Adesina, Emmanuel Donbraye, Moses Olubusuyi Adewumi

**Affiliations:** 1 Department of Microbiology, Faculty of Science, Obafemi Awolowo University, Ile-Ife, Osun State, Nigeria; 2 Department of Medical Microbiology, College of Health Sciences, Obafemi Awolowo University, Ile-Ife, Osun State, Nigeria; 3 Department of Virology, College of Medicine, University College Hospital, University of Ibadan, Ibadan, Oyo State, Nigeria

**Keywords:** Hepatitis B, Transfusion, Serological markers, ELISA, Blood donors

## Abstract

**Background:**

Transfusion associated Hepatitis B virus (TAHBV) continues to be a major problem despite mandatory screening for Hepatitis B surface Antigen (HBsAg). Presence of HBsAg is the common method for detecting hepatitis B infection. Unfortunately, this marker is not detected during the window period of the infection. Nigeria being a developing country cannot afford DNA testing of all collected units of blood which serve as the only possibility of achieving zero risk of transfusion associated HBV. Five different serological makers of hepatitis B virus (HBV) infection were therefore assessed to evaluate the reliability of using HBsAg marker alone in diagnosis of HBV infection among blood donors and to detect the serological evidence of the infection at the window period. This will preclude the possibility of transmitting hepatitis B through transfusion of Hepatitis B surface antigen (HBsAg) negative blood in Nigeria.

**Methods:**

Between July and August 2009, 92 blood donors were enrolled for the study. The prevalence of 5 different markers of Hepatitis B virus infection was detected using Enzyme Linked Immunosorbent Assay (ELISA). Demographic factors were assessed during the study.

**Results:**

HBsAg and its antibody (anti-HBs) was detected in 18 (19.6%) and 14(15.2%) of the 92 blood donors respectively. Anti-HBc IgM was found in 12(13.0%) of the 92 blood donors while Hepatitis B envelope antigen (HBeAg) and its antibody (anti-HBe) were detected in 4(8.9%) and 12(26.7%) respectively from 45 donors sampled. HBeAg is a marker of high infectivity and appears after HBsAg. At least one serological marker was detected in 30(32.6%) of the blood donors. Five (5.4%) of the 92 donors had anti-HBc IgM as the only serological evidence of hepatitis B virus infection.

**Conclusions:**

The result of this study shows that five donors have anti-HBcIgM as the only serological evidence of HBV infection. Inclusion of anti-HBcIgM in routine screening of blood donors in Nigeria should be encouraged. This is the first study to assess anti-HBcIgM in the country.

## Background

Hepatitis B virus (HBV) infection with its associated sequel is a disease of major public health importance, being the 10^th ^leading cause of death globally [1,2]. HBV infection accounts for 500,000 to 1.2 million deaths each year [3]. Of the approximately 2 billion people infected worldwide, more than 350 million are chronic carriers of HBV [4]. Approximately 15-40% of infected patients will develop cirrhosis, liver failure or hepatocellular carcinoma (HCC) [5,6]

The aetiological agent (Hepatitis B virus) is a member of the family Hepadnaviridae and the genus Orthohepadnavirus [7]. It is a double stranded circular DNA virus composed of an outer envelope containing hepatitis B surface antigen (HBsAg) and an inner nucleocapsid consisting of hepatitis B envelope antigen (HBeAg) and hepatitis B core antigen (HBcAg). Corresponding antibodies to each of these antigens are Hepatitis B surface antibody (anti-HBs or HBsAb), Hepatitis B envelope antibody (anti-HBe or HBeAb) and hepatitis B core IgM and IgG antibody (anti-HBc or HBcAb) [8]. The viral core also contains double stranded DNA genome and DNA polymerase. The serological markers of Hepatitis B virus are HBsAg, anti-HBs, HBcAg, anti-HBc (IgM and IgG), HBeAg, anti-HBe, and HBV DNA; these are important as they can be used in the diagnosis of the infection and to determine the severity of the infection [9].

Following infection by the hepatitis B virus (HBV), the first serological marker to appear in the blood is the HBV DNA, followed by HBsAg, the DNA polymerase and HBeAg. Thereafter, the antibodies to the hepatitis B antigens (HBcAb, HBeAb and HBsAb) can be detected. Screening of donated blood by enzyme-linked immunosorbent assay (ELISA) for HBsAg is the common method for detecting hepatitis B infection [10]. Screening of blood for the detection of this viral marker, however, does not rule out the risk of transmission of hepatitis B totally, because during the host serological response to infection, there is a phase during which the HBsAg cannot be detected in the blood although hepatitis B infection is present. This phase is known as the 'window period'. It represents a carrier state of the disease.

Findings indicate that testing done for HBsAg alone is not sufficient to eliminate HBV infection from blood supply [10-13]. A marker which would be indicative of hepatitis B infection during the window period is thus of paramount importance in blood banking especially in low income country like Nigeria, where DNA testing of all collected units of blood is not feasible.

This study was therefore designed to evaluate the reliability of using HBsAg marker alone, in diagnosis of HBV infection among blood donors and to detect the presence of a marker which would be indicative of hepatitis B infection during the window period.

## Results and Discussion

### Results

#### Prevalence of HBV Marker

Of the 92 blood samples tested, HBsAg was detected in 18 (19.6%), anti-HBs in 14(15.2%) and anti-HBcIgM in 12(13.0%). HBeAg and anti-HBe were detected in 4(8.9%) and 12(26.7%) respectively from 45 donors sampled. Table 1 summarises the frequency of the 5 markers of HBV tested among the blood donors. At least one serological marker was detected in 30(32.6%) of the blood donors (Table 2). Five (5.4%) of the donors had anti-HBcIgM as the only serological evidence of hepatitis B virus infection.

**Table 1:** Prevalence of Hepatitis B Virus Serological markers among blood donors

SEROLOGICAL MARKERS	NUMBER OF SAMPLES	POSITIVE (%)
HBsAg	92	18(19.6)
HBsAb	92	14(15.2)
HBeAg	45	4(8.9)
HBeAb	45	12(26.7)
HBcIgM	92	12(13.0)

**Table 2:** Donors showing detectable evidence of HBV infection by presence of at least one serological marker

S/N	Sample code	Age	Sex	SEROLOGICAL MARKERS				
				HBsAg	HBsAb	HBcIgM	HBeAg	HBeAb
1	111	30	M	✓		✓	✓	
2	117	23	M	✓			✓	
3	137	19	M	✓			✓	
4	138	41	M	✓			✓	
5	79	45	M	✓	✓			✓
6	80	37	M	✓	✓			✓
7	112	29	M	✓	✓			✓
8	113	22	M		✓			✓
9	121	22	M	✓				✓
10	124	35	M		✓			✓
11	153	19	M		✓			✓
12	154	21	M	✓		✓		✓
13	155	20	M		✓			✓
14	157	28	M	✓		✓		✓
15	158	32	M		✓	✓		✓
16	116	23	M		✓	✓		✓
17	123	27	M			✓		
18	163	33	M		✓	✓		
19	165	23	M			✓		
20	189	22	M			✓		
21	203	26	M	✓		✓		
22	15-5	29	M			✓		
23	04	21	M			✓		
24	07	27	F	✓				
25	08	36	M	✓	✓			
26	210	23	M	✓	✓			
27	215	19	F	✓				
28	69	20	F	✓	✓			
29	190	21	M	✓				
30	124	28	M	✓	✓			
TOTAL				18	14	12	4	12

#### Sex and Age distribution of donors with HBV marker

Of the 69 male blood donors enrolled for this study, 15 (21.7%) 13(18.8%) and12 (17.4%) were positive for HBsAg, HBsAb and Anti-HBcIgM respectively while 4(8.9%) and 12 (26.7%) were positive for HBeAg and HBeAb respectively among the 45 donors tested. Among the 23 females enrolled in the study no positivity was recorded for HBcAb, HBeAg and its Ab, however, 3(13%) and 1(4.3%) were positive for HBsAg and Ab respectively. (Table 3). There is no significant difference (P < 0.005) between the sex distribution of HBV markers among blood donors. The age distribution of blood donors with serological marker is shown in table 4.

**Table 3:** Sex distribution of blood donors with HBV markers

	Number Tested (%)	Positive (%)				Positive (%)	
		HBsAg	HBsAb	Anti-HBcIgM	No tested for HBeAg &Ab	HBeAg	HBeAb
Male	69(75%)	15 (21.7)	13 (18.8)	12 (17.4)	45	4 (8.9)	12(26.7)
Female	23(25%)	3 (13.0)	1 (4.3)	0 (0)	45	0 (0)	0 (0)
Total	92	18	14	12	45	4	12

**Table 4:** Age distribution of blood donors with Hepatitis B Virus serological markers

		Positive (%)				Positive (%)	
Age	No tested	HBsAg	HBsAb	HBcIgM	No tested	HBeAg	HBeAb
18-23	49	8(16.3)	6(12.2)	4(8.2)	19	2(10.5)	6(31.6)
24-29	18	5(27.8)	2(11.1)	4(22.2)	6	0(0)	2(33.3)
30-35	13	1(7.7)	3(23.1)	3(23.1)	10	0(0)	2(20.0)
36-41	10	3(30.0)	2(20.0)	1(10.0)	8	1(12.5)	1(12.5)
42-47	2	1(50.0)	1(50.0)	0(0)	2	0(0)	1(50.0)
TOTAL	92	18(19.6)	14(15.2)	12(13.0)	45	4(8.9)	12(26.7)

## Discussion

Transfusion of blood collected from a donor who is in the window period may lead to post transfusion hepatitis B in the recipient [14,15]. The result of this study showed that 5 donors (5.4%) had anti-HBcIgM antibody as the only serologic evidence of HBV. They have not seroconverted to anti-HBs, hence might be infectious. Previous studies have demonstrated the presence of HBV DNA in anti-HBc-only donors [16,17]. These findings corroborate earlier report that testing blood donors for HBsAg alone is not sufficient to eliminate HBV from blood supply [11]

Chaudhuri et al., 2003 observe the presence of HBV DNA in one-fifth of anti-HBc-only donors studied and stated that, reactivity to anti-HBc only can predict cryptic HBV infection. A report from Japan [16] demonstrated the presence of HBV DNA in 38 percent of HBsAg-negative anti-HBc-reactive donors. The only serologic evidence of infection with hepatitis B in 5 of the 92 blood donors in this study is the presence of IgM antibody to the core antigen (HBcIgM). Blood from the 5 anti-HBc IgM only donors from this study might have been transfused to innocent patients (since the donors were screened negative due to no detectable HBsAg in their blood), thereby increasing the number of possible post transfusion HBV.

It had been observed earlier [10,14,15] that the screening test for detection of HBsAg does not rule out the transmissions of Hepatitis B as the donor might be in the "window period" and detection of the antibody to the hepatitis B core antigen HBcIgM type serves as a useful serological marker during this period. A prevalence of 5.4% observed for anti-HBc in this study is higher compared with 0.39% observed by Col *et al.*, [10]. This might be indicating a progressive increase in the possibility of transfusing HBV through blood donation.

Anti-HBc has been found to be an excellent indicator of occult HBV infection during the window period [18,19,15]. Other markers for detecting occult HBV infection in an HBsAg negative blood donor such as detection of HBV DNA by polymerase chain rection (PCR) may not be cost effective [20]). Detection of anti-HBc has contributed significantly in reducing the incidence of post transfusion hepatitis B amongst patients [21-23]. The IgM class of the anti-HBc is a marker that indicates recent infection. The IgG variety of anti-HBc appears later during the infection and points to a past HBV infection. Individuals with IgG variety of anti-HBc may not be infectious as they may have sufficiently high titres of antibodies to HBsAg (anti-HBs), which are protective in nature and the affected individuals may actually be disease free. The sero-prevalence rate of anti-HBcIgM in the general population in Nigeria is not available. To the best of our knowledge, the only published data on estimation of anti-HBc in Nigeria was on the prevalence of anti-HBc total (i.e. both IgG and IgM) amongst patients with chronic liver disease and it was estimated as 90% [24]. Anti-HBcIgG may remain positive for life in an infected individual although the individual has protective levels of anti-HBs and therefore does not necessarily mean that blood of such a donor is infectious. Consequently, anti-HBcIgM is considered to be a more specific marker for HBV infection during the window period [25-27].

## Conclusion

The result of this study shows the need to include anti-HBcIgM in routine screening of blood donors in Nigeria. It also confirms the fact that testing blood donors for HBsAg alone is not sufficient to eliminate HBV from blood supply. Although, the possibility of achieving zero risk of transfusion associated HBV infection depend largely on DNA testing of all the collected units of blood [13] before transfusion; however, since this is not done in many developing countries including Nigeria due to cost, this study recommends the inclusion of anti-HBcIgM in routine screening of blood donors in countries where DNA testing is not done. This will go a long way in reducing transfusion associated Hepatitis B Virus (TAHBV) infection.

## Methods

### Sample Collection and Preparation

Ninety-two (M = 69, F = 23; Age range: 18-50 years; Mean: 24.9 years) blood donors in Ile-Ife, Osun State were enrolled for the study between July and August 2009. Five millilitres of blood was collected from each donor by venepuncture into a labelled sterile container free of anticoagulants or preservative. Each blood specimen was separated by low centrifugation at 500 g for 5 minutes and the serum transferred into labelled cryovial. Thereafter, the sera were kept frozen at -20C until analysed.

### Assay procedure

Five different markers of HBV infection (HBsAg, anti-HBs, anti-HBcIgM, HBeAg and anti-HBe) were detected by commercial Enzyme-linked immunosorbent assay (ELISA) kit (Diapro Diagnotic Bioprobes Milano- Italy). The kit, reagent and sera were brought to room temperature before starting the assay. The wells of each of the microplate were identified for controls and samples. For each of the assays, there were two negative controls and one positive control. One hundred microlitres each of negative control, positive control and sample were pipetted into the appropriate wells except for HBsAg where 50 μl was used as stated by the manufacturer. The plates were incubated at 37°C for 60 minutes, washed with wash buffer 5 times with soaking time of about 30 seconds in between each washing. A second incubation at same temperature and time was carried out after the addition of enzyme conjugate and the plates were washed as previously described. A final incubation at room temperature for 10 to 15 minutes depending on manufacturer's instruction for the specific serological marker was done after the addition of substrate (a chromogen). This made the positive samples turn to blue while the negatives were colourless. Addition of 100 μl of the stop solution (sulphuric acid) turned the positive samples from blue to yellow. The results were read using a 450 nm filter microplate reader.

### Statistical analysis

The prevalence of serological markers was compared in terms of gender by use of chi-square tests.

## List of Abbrevations

Anti-HBcIgM: Hepatitis B Core IgM Antibody; HBsAg: Hepatitis B surface Antigen; TAHBV: Transfusion Associated Hepatitis B Virus; HBV: Hepatitis B Virus; ELISA: Enzyme Linked Immunosorbent Assay; Anti-HBs or HBsAb: Hepatitis B surface Antibody; HBeAg: Hepatitis B envelope Antigen; Anti-HBe or HBeAb: Hepatitis B envelope antibody.

## Competing interests

The authors declare that they have no competing interests.

## Authors' contributions

MOJ conceived the study. MOJ and OAA were in charge of recruitment of patients and collection of samples. MOJ, OAA and ED undertook laboratory analysis; MOJ analyzed the data and prepared the manuscript; OAA and MOA provided ideas and comments during manuscript preparation. All authors have read through and approved the final manuscript.
